# ORAL HEALTH AMONG FILIPINO OLDER ADULT IMMIGRANTS

**DOI:** 10.1093/geroni/igad104.3510

**Published:** 2023-12-21

**Authors:** Emerson Ea, Bei Wu, Jinyu Hu

**Affiliations:** New York University, New York City, New York, United States; New York University, New York City, New York, United States; New York University, New York City, New York, United States

## Abstract

Poor oral health has been associated with decreased quality of life, various non-communicable diseases, and a higher risk of mortality. There is also evidence that older immigrants and those who have been in the US for less than 5 years are more likely to have periodontitis, a serious gum disease and a common oral health condition. Despite this evidence, there is a paucity of studies investigating the influence of acculturation, immigration-related experiences, and resilience on oral health among older Filipino immigrants. To address this significant knowledge gap, a mixed methods study using a survey and focus group interviews was conducted among community-dwelling Filipino immigrants aged 60 years and older. This poster will present the results of the survey (N=103), which used a socio-demographic questionnaire, as well as valid and reliable instruments such as the Geriatric Oral Health Assessment Index, A Short Acculturation Scale for Filipino Americans, Everyday Discrimination Scale, and Connor-Davidson Resilience Scale. The results of the regression analysis revealed that the perception of discrimination and resilience were significantly associated with the perception of oral health among the participants surveyed [F(10,92) = 1.985, p < 0.05]; this regression model accounted for 18.6% of variance in the perception of oral health. The findings support the need for culturally sensitive interventions that could optimize oral health, including the utilization of dental care services for this underserved population.

